# PKC-δ mediates interferon-α-induced apoptosis through c-Jun NH_2_-terminal kinase activation

**DOI:** 10.1186/1471-2121-13-7

**Published:** 2012-03-21

**Authors:** Noriko Yanase, Miho Hayashida, Yuki Kanetaka-Naka, Akinori Hoshika, Junichiro Mizuguchi

**Affiliations:** 1 Department of Immunology and Intractable Immune System Disease Research Center, Tokyo Medical University, 6-1-1 Shinjuku, Shinjuku-ku, Tokyo 160-8402, Japan; 2 Department of Pediatrics, Tokyo Medical University Hospital, 6-7-1 Nishi-shinjuku, Shinjuku-ku, Tokyo 160-0023, Japan

**Keywords:** Interferon-α, PKC-δ, JNKs, Apoptosis, B lymphoma cells

## Abstract

**Background:**

Interferon-α (IFN-α) exerts an anti-tumor effect at least through induction of apoptosis in a variety of types including B lymphoma cells. We recently found that IFN-α induced a sustained activation of c-Jun NH_2_-terminal kinase1 (JNK1), which is implicated in activation of the tumor necrosis factor (TNF)-related apoptosis-inducing ligand (TRAIL) promoter. In the present study, we explored upstream component(s) of the prolonged IFN-α-initiated activation of JNK1.

**Results:**

IFN-α caused activation of PKC-δ in Daudi B lymphoma cells and myeloma U266 cells, as detected by Western blotting using a monoclonal antibody specific for the phosphorylated form of PKC-δ. The dominant-negative form of mutant PKC-δ (dnPKC-δ) reduced the IFN-α-induced JNK1 activation, TRAIL promoter activity, loss of mitochondrial membrane potential (ΔΨm), and increase in propidium iodide (PI) positive cells. The IFN-α-induced activation of JNK1 and the TRAIL promoter was also attenuated by the PKC-δ inhibitor rottlerin. Moreover, a constitutively active form of mutant PKC-δ enhanced the IFN-α-induced TRAIL promoter activity and loss of ΔΨm in Daudi B lymphoma cells. In addition, IFN-α-induced Ser727 phosphorylation of Stat1 was also abrogated by dnPKC-δ.

**Conclusions:**

IFN-α induced JNK1 activation via PKC-δ, leading to upregulation of TRAIL. The interaction of the consequent enhanced TRAIL expression with TRAIL-receptor results in a loss of ΔΨm and increase in PI positive cells. The IFN-α-induced apoptotic events may also be affected by the Ser727-Stat1 induced by PKC-δ-mediated signaling component(s).

## Background

Type I interferon-α (IFN-α) has been employed for treatment of patients with some tumors including hairy cell leukemia, chronic myelogenous leukemia, melanoma, and renal cell cancer [1,2]. The anti-tumor action of IFN-α is mediated at least by induction of apoptosis or inhibition of cell growth [3-6]. However, the detailed molecular mechanism(s) by which IFN-α induces apoptosis remain largely unclear. The interaction of IFN-α with its receptor results in transphosphorylation of receptor-associated Janus kinases (Jak1 and Tyk2), leading to tyrosine phosphorylation of critical residues on the receptors [7,8]. The activated Jak1/Tyk2 phosphorylate and activate signal transducers and activators of transcription (STAT), which in turn translocates to the nucleus to regulate gene transcription [8].

In addition to Jak/Stat pathways, mitogen-activated protein kinases (MAPKs) including extracellular kinase 2 (ERK2), p38MAPKs, and c-Jun NH_2_-terminal kinases (JNKs) are activated in response to IFN-α in some cell types including B lymphoma cells [5,9-12]. For instance, IFN-α induced prolonged JNK1 activation [5,12] with decreased ERK activity, resulting in dysfunction of mitochondrial membrane potential (ΔΨm). p38MAPK was also activated by IFN-α [13,14]. Recent studies have determined that MAPKs activated by IFN-α receptors (IFNARs) are downstream of several components including protein kinase C (PKC) [10,15]. The PKC family proteins are divided into three subclasses [16,17]: 1) atypical PKCs (ζ and λ), which are activated by phosphatidylserine; 2) conventional PKCs (α, β, and γ), which are calcium-dependent and activated by diacylglycerol; and 3) the novel PKCs (δ, ε, and θ), which are calcium-independent and activated by diacylglycerol. A novel ubiquitously expressed PKC, PKC-δ, is phosphorylated and activated in response to multiple agents including IFN-α [18-21]. The activated PKC-δ mediates apoptosis in some cell types and survival in other cell types [15,22,23].

In the present study, we examined whether PKC-δ is involved in the IFN-α-induced apoptosis in Daudi B lymphoma and U266 myeloma cells. Our data clearly indicate that IFN-α-induced PKC-δ signaling leads to activation of JNK1 and Stat1. These observations would be useful for the development of new treatment modalities of patients by using IFN-α

## Results

### IFN-α induces phosphorylation of PKC-δ in both daudi B lymphoma and U266 myeloma cells

PKC family proteins have multiple effects on both pro-apoptotic and anti-apoptotic functions [15,22,23]. The levels of PKC family proteins in Daudi B lymphoma cells were determined by Western blotting. Substantial levels of PKC-α, -δ, and -ι were detected in Daudi cells, with minute amounts of PKC-γ and -η (Hayashida et al., unpublished observations). PKC-ε and -λ were almost undetectable. Because PKC-δ exerts a pro-apoptotic effect in some cell types [15], we examined whether PKC-δ is involved in IFN-α-mediated apoptosis in human B lymphoma cells.

**Figure 1:**
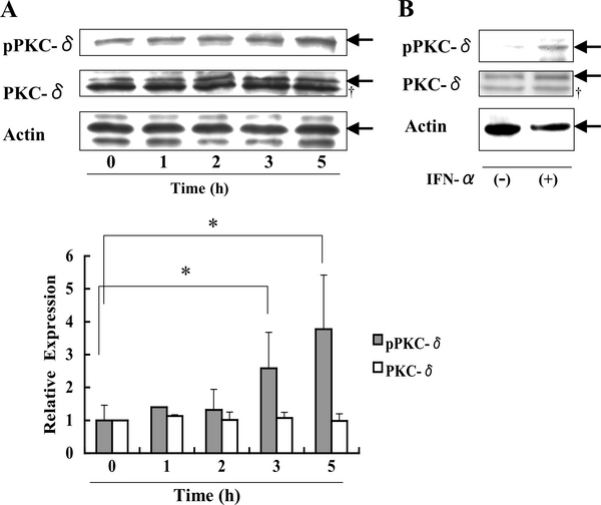
**IFN-α induces phosphorylation of PKC-δ in Daudi and U266 B lymphoma cells**. Daudi (A) and U266 (B) cells were cultured with 250 U/ml IFN-α, followed by Western blotting using Abs specific for pan- or phospho (Thr505)-PKC-δ. Arrows indicate specific bands for pPKC-δ, PKC-δ, and Actin, respectively. †, non-specific band. Expression of pan- or phospho-PKC-δ relative to actin is shown. Each point represents mean ± SD from five independent experiments. *Significantly different (*p *< 0.05) from time zero.

Daudi cells were stimulated with IFN-α for the indicated times, followed by assay by Western blotting using an antibody (Ab) specific for phospho-PKC-δ (Thr505) or total PKC-δ. In unstimulated Daudi cells, a certain level of PKC-δ phosphorylation was detected, which was increased up to 5 h following stimulation with IFN-α (Figure 1A). Similar to Daudi B lymphoma cells, IFN-α induced the PKC-δ phosphorylation in the myeloma cell line U266 (Figure 1B). These results suggest that IFN-α causes PKC-δ phosphorylation in human B lineage cells.

### PKC-δ inhibitor rottlerin prevents IFN-α-induced PKC-δ activation

**Figure 2:**
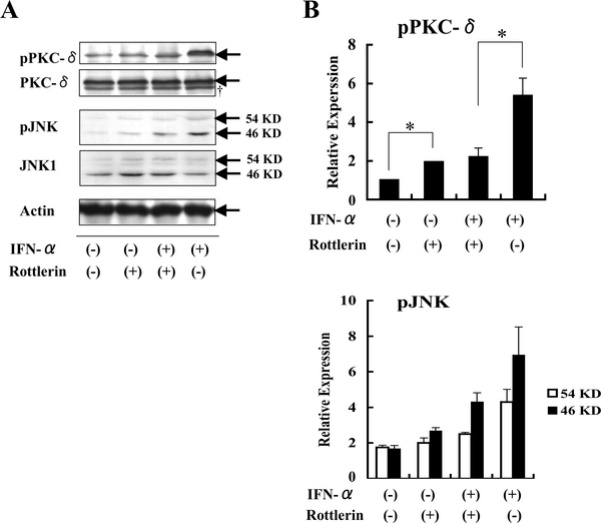
**PKC-δ inhibitor rottlerin abrogates IFN-α-induced phosphorylation of PKC-δ**. Daudi cells pretreated with 1 μM rottlerin for 1 h were stimulated with 1,000 U/ml IFN-α for 6 h, followed by assays for levels of phospho-PKC-δ and phospho-JNK (A). Arrows indicate specific bands for pPKC-δ, PKC-δ, pJNK, JNK1, and Actin, respectively. †, non-specific band. Results are expressed as relative expression: phospho-PKC or phospho-JNK levels normalized to actin levels with the indicated stimulators are divided by phospho-PKC-δ or phospho-JNK levels normalized to actin levels without stimulation (B). Each point represents mean ± SD from three independent experiments (B&C). *Significantly different (*p *< 0.05) from control without inhibitor.

To examine whether PKC-δ is implicated in the IFN-α-induced activation of JNK, the PKC-δ inhibitor rottlerin was employed. Pretreatment of Daudi cells with 1 μM rottlerin for 1 h substantially reduced the IFN-α-induced PKC-δ activation (Figure 2A &2B), while rottlerin alone induced a small, but significant level of PKC-δ activation, as assessed by Western blotting using mAbs specific for phospho-PKC-δ and total PKC-δ. Pretreatment with rottlerin showed a tendency to reduce the IFN-α-induced activation of JNK, although the difference was not statistically significant, as revealed by short (46 kDa) and long (54 kDa) forms of pJNK (Figure 2A and 2B). These results further suggest that IFN-α induces PKC-δ activation.

### PKC-delta inhibitor rottlerin reduces IFN-α-induced activation of TRAIL promoter

**Figure 3:**
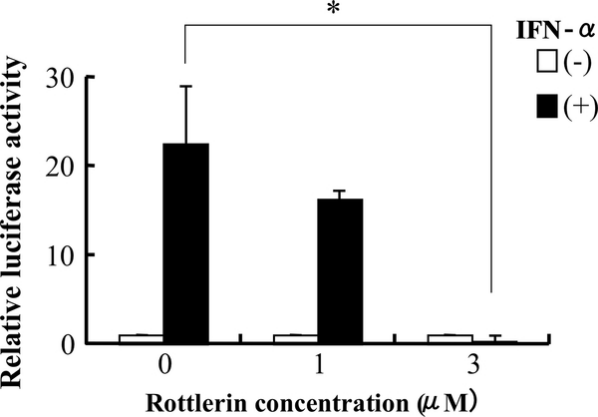
**PKC-δ inhibitor rottlerin abrogates IFN-α-induced activity of TRAIL promoter in Daudi cells**. Daudi cells were transfected with a TRAIL-Luc reporter, followed by incubation with medium alone for 24 h. The cells were pretreated with rottlerin for 1 h, stimulated with 1,000 U/ml IFN-α for 24 h, and then assayed for luciferase activity. Reporter activity was normalized relative to internal control Renilla luciferase values. Normalized data from experimental group are expressed as relative luciferase activity compared with those seen in control group. Each point represents mean ± SD from three independent experiments. *Significantly different (*p *< 0.05) from control without inhibitor.

We have previously demonstrated that JNK signaling is involved in the IFN-α-induced upregulation of TRAIL promoter activity [12]. Pretreatment with rottlerin prevented the IFN-α-induced TRAIL promoter activity in a dose-dependent manner, as determined by TRAIL-Luc assays (Figure 3). Thus, it is proposed that IFN-α induces PKC-δ signaling upregulates TRAIL promoter activity through JNK activation.

IFN-α-induced mitochondrial membrane potential and apoptosis are reduced in Daudi cells overexpressing dominant-negative PKC-δ, while it is partially enhanced in those overexpressing constitutively active PKC-δ

**Figure 4:**
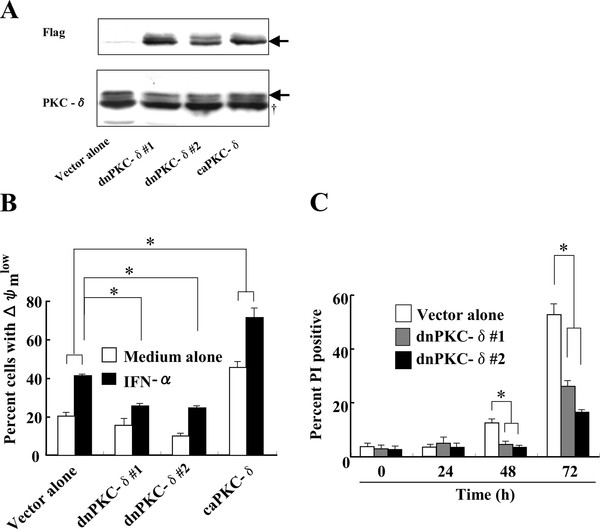
**IFN-α-induced loss of mitochondrial membrane potential and apoptosis are partially abrogated in dnPKC-δ-expressing cells, while somewhat enhanced in caPKC-δ-expressing cells**. Daudi cells were transfected with Flag-dnPKC-δ, Flag-caPKC-δ, or the control vector alone, and incubated with selection media containing hygromycin B, followed by limiting dilution to obtain clone(s). The resulting clones were confirmed to express Flag-dnPKC-δ or Flag-caPKC-δ (A). These clones were stimulated with 1,000 U/ml IFN-α for the indicated time periods, followed by assays for mitochondrial membrane potential (B, 48 h-incubation) and PI positive cells (C). Arrow indicates specific band for PKC-δ. †, non-specific band. The results from three independent experiments (B&C) are represented as mean ± SD. *Significantly different (*p *< 0.05) from control vector alone.

Rottlerin has some deleterious effects on mitochondrial function (Additional file 1, [24]), which was further aggravated by the addition of IFN-α. To further confirm that PKC-δ signaling is required for IFN-α-induced apoptosis in Daudi cells, dominant negative (dn) and constitutively active (ca) forms of mutant PKC-δ were employed [12]. Daudi cells were transfected with dnPKC-δ, caPKC-δ, or the control vector alone, followed by incubation with a medium containing hygromycin B. The resultant clones overexpressing dnPKC-δ (dnPKC-δ#1, dnPKC-δ#2) or caPKC-δ, but not the control, were confirmed to express species of the expected molecular weight, as detected by Western blotting using anti-Flag mAb (Figure 4A).

The cell lines overexpressing dnPKC-δ (#1 and #2) showed a reduced loss of ΔΨm and percentage of PI positive cells after IFN-α stimulation (Additional file 2 and Figure 4B and 4C), while the cells expressing caPKC-δ showed an enhanced loss of ΔΨm (Figure 4B). Even in the unstimulated condition, caPKC-δ-overexpressing cells exhibited an increased loss of ΔΨm compared with control vector alone, suggesting that PKC-δ functions as a pro-apoptotic molecule in the IFN-α-induced apoptosis in Daudi B lymphoma cells.

### IFN-α-induced activation of JNK and TRAIL promoter is partially reduced in daudi cells overexpressing dnPKC-δ

**Figure 5:**
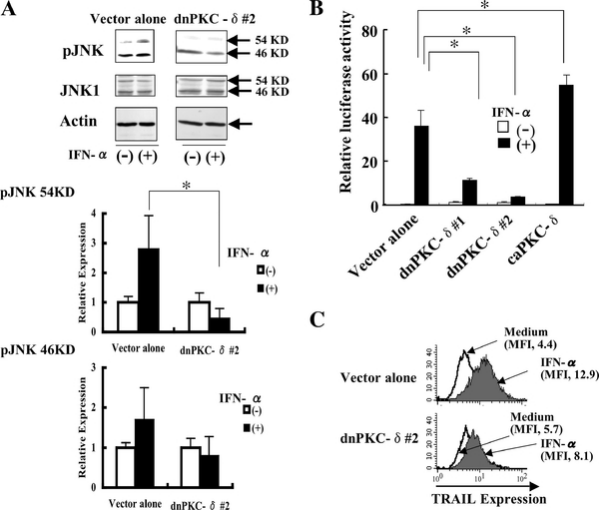
**IFN-α-induced activation of JNK1 and TRAIL promoter is partially reduced in dnPKC-δ-expressing cells**. The dnPKC-δ-expressing Daudi cells were stimulated with 1,000 U/ml IFN-α for 6 h, and the levels of phospho-JNK (A), TRAIL promoter activity (B), and TRAIL protein expression on the cell surface (C) were determined by Western blotting, the TRAIL-Luc reporter assay, and flow cytometry, respectively. MFI represents mean fluorescent intensity. The results from three independent experiments (B) are represented as mean ± SD. *Significantly different (*p *< 0.05) from control vector alone.

The activities of JNK and the TRAIL promoter in response to IFN-α were examined in dnPKC-δ-overexpressing cells. The IFN-α-induced enhancement of the phosphorylated form of JNK was not detected in the dnPKC-δ-expressing cells, compared with the control vector alone (Figure 5A). The IFN-α-induced TRAIL promoter activity was decreased and increased in dnPKC-δ- and caPKC-δ-expressing cells, respectively, compared with the control vector alone (Figure 5B). Flow cytometric analysis showed that the IFN-α-induced increase in TRAIL protein expression on dnPKC-δ-expressing cells was low, compared with control (Figure 5C). Moreover, JNK inhibitor SP-600125 reduced the IFN-α-induced TRAIL promoter activity in the caPKC-δ-expressing cells (Additional file 3). These findings suggest that IFN-α-induced PKC-δ signaling results in JNK activation, leading to TRAIL promoter activation.

### IFN-α-induced phosphorylation ser-727 of Stat1 is substantially reduced in dnPKC-δ-expressing cells

**Figure 6:**
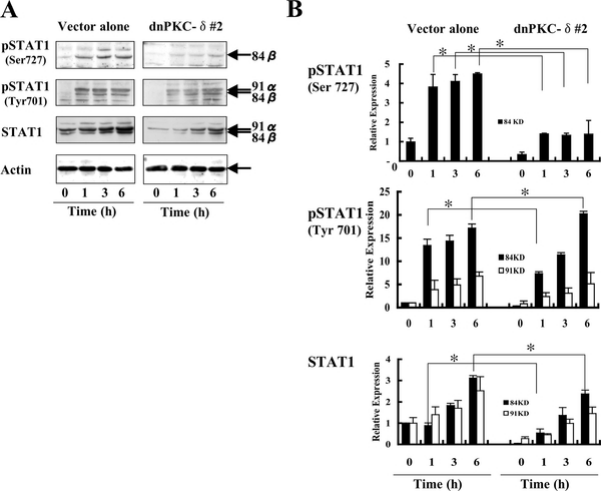
**IFN-α-induced Ser727 phosphorylation of Stat1 is substantially abrogated in the dnPKC-δ-overexpressing cells**. The cells overexpressing dnPKC-δ or the control vector alone were stimulated with 1,000 U/ml for the indicated time periods, followed by assays for Ser727 and Tyr701 phosphorylation of Stat1 using Abs specific for phospho-Ser727 and phospho-Tyr701, respectively (A). Results are expressed as relative expression (B), as shown in Figure 2. *Significantly different (*p *< 0.05) from control vector alone.

IFN-α is known to phosphorylate Stat1 on the Ser727 residue in human U266 myeloma and human fibroblast cell line [19,25]. The IFN-α-induced phosphorylation (Ser727) was considerably prevented in the dnPKC-δ-expressing cells relative to control cells (Figures 6A and 6B). Levels of total Stat1 were increased in both the dnPKC-δ-expressing and the control cells. However, Stat1 phosphorylation at tyrosine 701 was detected at almost comparable levels between dnPKC-δ-expressing and control cells 6 h after stimulation with IFN-α. Together, IFN-α-induced PKC-δ signaling regulates JNK activation and Ser727-Stat1 phosphorylation, which might affect apoptotic processes.

## Discussion

IFN-α has been employed for treatment of tumors and viral diseases (hepatitis C) [1,2]. Type I IFN-α exerts multiple functions including anti-viral action, immune modulation, cell proliferation, and cell death [3,4,26-28]. IFN-α actions against tumors involve induction of apoptosis as well as indirect effects through induction of cytotoxic cells [7,29]. We have previously demonstrated that IFN-α induced a sustained activation of pro-apoptotic molecule JNKs [12], concomitant with upregulation of anti-apoptotic molecule c-Flip through NF-κB activation [30]. However, the molecular mechanisms of IFN-α-induced apoptotic cell death are not completely understood. In the present study, we explored the upstream component of IFN-α-induced JNK activation and found that PKC-δ activation occurs upstream of JNK and Stat1 activation.

Several studies have demonstrated that PKC-δ has multiple targets in response to apoptotic stimuli, including IFN-α [15,22,23]. For example, it has been shown that PKC-δ mediates JNK activation in response to DNA damage [18]. Consistent with this observation, genetic silencing of PKC-δ using dnPKC-δ reduced the IFN-α-induced JNK activation (Figure 5A), supporting the notion that PKC-δ signaling participates in the IFN-α-induced JNK activation. Because PKC-δ did not translate to the nucleus in response to IFN-α (Additional file 4), it is unlikely that PKC-δ affects gene expression via transcription factor. Rather, downstream component of PKC-δ signaling pathway including JNK may participate in the upregulation of TRAIL promoter activity [12].

The interaction of TRAIL with its receptors TRAIL-Rs leads to caspase-8 activation through recruitment of adaptor protein Fas-associated death domain (FADD) [31]. The caspase-8 activation in most cell types is amplified in the mitochondria. The IFN-α-induced loss of ΔΨm and increase in sub-G1 fraction was reduced in the dnPKC-δ-overexpressing cells, while the mitochondrial dissipation was enhanced in the caPKC-δ-overexpressing cells. PKC-δ inhibitor rottlerin was not suitable for analysis of IFN-α-induced loss of ΔΨm in Daudi cells, because rottlerin alone induced mitochondrial dissipation (Additional file 1, [24,32]). Such rottlerin-induced mitochondrial damage may lead to a small level of PKC-δ activation. Thus, the following pathway is proposed for the IFN-α-induced apoptosis: IFN-α induces JNK1 activation via PKC-δ → upregulation of TRAIL → interaction of TRAIL with its TRAIL-Rs. → loss of ΔΨm → apoptosis. Interestingly, a recent report clearly demonstrated that TRAIL induces PKC-δ activation through diacylglycerol in Jurkat cells [33], which can form a positive feedback loop involving PKC-δ and TRAIL. Alternatively, it is also possible that the activated JNK induces migration of p21Bax-α to the mitochondria from cytosol, which is cleaved into p18Bax-α, a more potent form [34,35]. These changes lead to loss of ΔΨm, especially in the later phases of apoptotic processes, which is TRAIL-independent [36].

The TRAIL promoter contains a possible IFN-stimulated response element (ISRE) and AP-1 sites [37-39], which might be involved in TRAIL promoter activation. Originally, JNK was found to induce phosphorylation of c-Jun, which combines with c-Fos, creating a heterodimer of c-Jun/c-Fos (AP-1) [10]. Indeed, IFN-α caused AP-1 activation in several cell lines including Daudi lymphoma cells, probably through JNK activation (Yanase et al. ms. in preparation, [12]). The ISRE binds transcription factor IFN-stimulated gene factor-3 (ISGF3) comprising Stat1, Stat2, and IRF-9 [7]. IFN-α caused phosphorylation of Stat1 on Ser727, at least through PKC-δ signaling [19,21]. We also confirmed the phosphorylation of Stat1 (Ser727) in the IFN-α-stimulated Daudi cells (Figure 6A). The IFN-α-stimulated phosphorylation of Stat1 on Ser727, but not Tyr701, was markedly abrogated in the dnPKC-δ-expressing Daudi cells, confirming that PKC-δ signaling plays a crucial role in the Ser727-PKC-δ phosphorylation. Stat1 activation favours induction of apoptosis in some settings, whereas Ser727-Stat1 promotes survival in others probably through gene induction [40-42]. Experiments are required to address whether IFN-α-induced Ser727-Stat1 functions as proapoptotic or anti-apoptotic molecule.

Several kinases phosphorylate and activate PKC-δ following stimulation with IFN-α. For example, tyrosine phosphorylation of PKC-δ on Tyr 311 was induced by Src-tyrosine kinase [43,44]. Interestingly, PI3-K and phosphoinositide-dependent kinase (PDK)1 [45] phosphorylated PKC-δ on the Thr505 residue that is stimulated by IFN-α (Figure 1). It remains unclear what kinases are involved in IFN-α-stimulated phosphorylation of PKC-δ in Daudi B lymphoma cells.

Stimulation of IFN-α together with chemotherapeutic agents appears to be effective for treatment of cancer patients [46,47]. PKC-δ-JNK signaling pathway is involved in induction of apoptosis in response to chemotherapeutic agents [18]. Here, we demonstrated that the IFN-α-induced apoptosis also involves PKC-δ-JNK signaling pathway in human Daudi B lymphoma cells. Concurrent with the activation of pro-apoptotic pathway, we recently demonstrated that IFN-α-induced-PKC-α activation caused upregulation of anti-apoptotic c-Flip through NF-κB activation [30]. Thus, the balance between IFN-α-induced pro-apoptotic (such as PKC-δ-JNK) and anti-apoptotic (such as PKC-α-NF-κB) signaling pathways might determine the cell phenotype. These findings would be valuable for the design of treatment modalities of cancer patients with IFN-α together with or without chemotherapeutic agents.

## Conclusions

IFN-α is known to induce JNK activation leading to upregulation of TRAIL promoter activity. In the present study, we explored the upstream component of JNK activation and found that IFN-α induced PKC-δ signaling caused JNK activation, resulting in induction of TRAIL-mediated apoptosis. In addition, the IFN-α-induced PKC-δ signaling was also involved in Stat1 phosphorylation on serine 727, which may also affect the cell phenotype. These observations would be useful for the development of new treatment modalities of cancer patients with IFN-α.

## Methods

### Cell culture

Human Daudi B lymphoma and U266 myeloma cells were obtained from American Type Culture Collection (Manassas, VA, USA) and were maintained at 37°C in 5% CO_2 _in RPMI-1640 medium supplemented with 10% fetal bovine serum (FBS), 50 μM 2-mercaptoethanol, and 100 μg/ml kanamycin. The cells were cultured with or without PegIFN-alpha2a (IFN-α; 1.67 × 10^4 ^U/μg: Hoffmann-LaRoche, Basel, Switzerland) in the presence or absence of PKC-δ inhibitor rottlerin (Merck Millipore, Tokyo, Japan) for the indicated times. Cell lines overexpressing dnPKC-δ (K376A), caPKC-δ (DR144/145A), or the control vector alone [48] were established, according to the procedure previously described [12].

### Western blot analysis

Western blotting was performed as previously described [12]. Briefly, cells were solubilized in lysis buffer, and equivalent amounts of protein were separated on 8.5% SDS-PAGE, followed by transfer to a PVDF membrane. After several washes, the membranes were incubated with primary Abs: mouse anti-PKC-δ monoclonal Ab (mAb) (BD Transduction Labs., San Jose, CA, USA), mouse anti-Flag mAb (Sigma, St. Louis, MO, USA), rabbit anti-JNK1 Ab, rabbit anti-STAT1 Ab (Santa Cruz Biotechnology Inc., Santa Cruz, CA, USA), rabbit anti-phospho-PKC-δ (Thr505) Ab, rabbit anti-phospho-STAT1 (Tyr701) Ab (Cell Signalling Technology, Denver, MA,, USA), rabbit anti-phospho-STAT1 (Ser727) Ab (Merck Millipore), mouse anti-phospho-JNKs (Thy183/Tyr185) mAb, and rabbit anti-actin Ab (Sigma, St. Louis, MO, USA). The bound primary Abs were then incubated with horseradish peroxidase (HRP)-labeled goat anti-rabbit IgG Ab or HRP-labeled goat anti-mouse IgG Ab (Cappel MP Biochemical, Aurora, OH, USA). The membrane-bound HRP-conjugated Ab was visualized by enhanced chemiluminescence according to the manufacturer's recommendations (Amersham Life Science, Buckinghamshire, UK). The density of each band was measured with a densitometer (Image Master DTS, Amersham Pharmacia Biotech).

### Flow cytometric analysis of mitochondrial membrane potential and propidium iodide staining

The cells were stimulated with IFN-α for the indicated periods, and were assayed for surface expression of TRAIL, cells with ΔΨm^low^, and PI positive cells by flow cytometry, as previously described [12].

### Subcellular fractionation

Lysates from IFN-α-stimulated cells were separated into the cytosolic and nuclear fractions using Nuclear/Cytosol Fractionation Kit (Bio Vision, Mountain View, CA, USA).

### Transient transfection assay

TRAIL promoter activity was determined by a luciferase reporter vector containing a TRAIL promoter fragment, as previously described [12]. Briefly, cells were transfected with 4.5 μg reporter constructs together with 0.5 μg pRL-CMV-Renilla luciferase vector (Toyo Inki) using a Gene Pulser according to the manufacturer's instruction (Lonza Cologne, Koeln, Germany). After incubation with medium alone for 24 h, cells were stimulated with or without 1,000 U/ml IFN-α for an additional 24 h. In some experiments caPKC-δ-expressing cells were stimulated with IFN-α in the presence or absence of JNK inhibitor SP-600125 (Calbiochem, Darmstadt, Germany). The cells were assayed for luciferase activity with a Luciferase Reporter Assay System (Promega Co., Madison, WI, USA).

### Statistical analysis

Data are expressed as the mean ± SD for each group. Statistical significance was determined by Student's *t-*test.

## Authors' contributions

NY made a substantial contribution to experiments reported in Figures 1, 4, 5A, C, and 6. MH made a substantial contribution to experiments reported in Figures 2 and 3. YK made a contribution to experiments reported in Figure 5B. AH was responsible for design of experiments. JM supervised the studies and prepared manuscript. All authors made considerable contributions for conception and design of experiments, and also analysis and interpretation of data. All authors read and approved the final manuscript.

## Supplementary Material

Additional file 1 **Rottlerin induces loss of mitochondrial membrane potential with or without IFN-α**. Daudi cells were cultured with the indicated concentrations of rottlerin in the presence or absence of 1,000 U/ml IFN-α for 48 h, followed by assay for mitochondrial membrane potential. Click here for file

Additional file 2 **IFN-α-induced loss of mitochondrial membrane potential and apoptosis are partially abrogated in the dnPKC-δ-expressing cells, while somewhat enhanced in the caPKC-δ-expressing cells**. The dnPKC-δ-expressing and caPKC-δ-expressing cells were stimulated with 1,000 U/ml IFN-α for the indicated time periods and assayed as described in Figure legend 4. A, percent cells with low mitochondrial membrane potential; B, percent PI positive cells. Click here for file

Additional file 3 **JNK inhibitor SP-600125 reduces TRAIL promoter activity induced by IFN-α in caPKC-δ-overexpressing Daudi B lymphoma cells**. The caPKC-δ-overexpressing Daudi B lymphoma cells were pretreated with SP-600125 for 1 h, stimulated with 1,000 U/ml IFN-α for 24 h, and then assayed for luciferase activity. *Significantly different (*p *< 0.05) from IFN-α without JNK inhibitor. Click here for file

Additional file 4 **PKC-δ does not translocate to the nucleus upon IFN-α stimulation**. Cells were stimulated with or without 1,000 U/ml IFN-α for the indicated time periods. Cell lysates were fractionated into the cytosolic and nuclear fraction, as described in materials and methods. Samples were assayed for expression levels of total and phospho-PKC-δ by Western blotting. Click here for file
