# Supplementary figures and images for: PKC-δ mediates interferon-α-induced apoptosis through c-Jun NH2-terminal kinase activation
Source: BMC Cell Biol. 2012 Mar 21;13:7. doi: 10.1186/1471-2121-13-7 (PMC3353249; doi:10.1186/1471-2121-13-7)

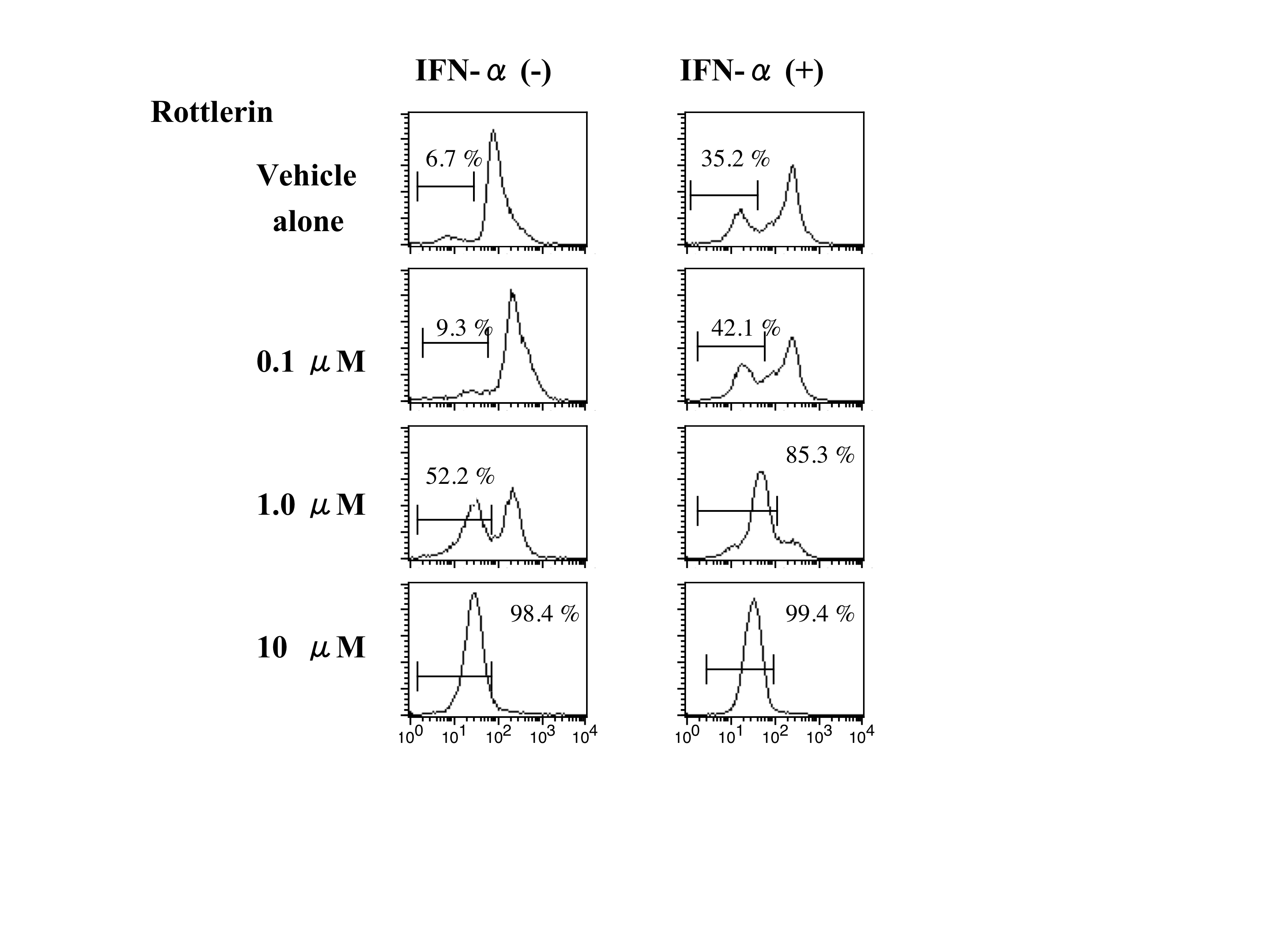

Supplement: Additional file 1 — Rottlerin induces loss of mitochondrial membrane potential with or without IFN-α. Daudi cells were cultured with the indicated concentrations of rottlerin in the presence or absence of 1,000 U/ml IFN-α for 48 h, followed by assay for mitochondrial membrane potential. [file 1471-2121-13-7-S1.PNG]

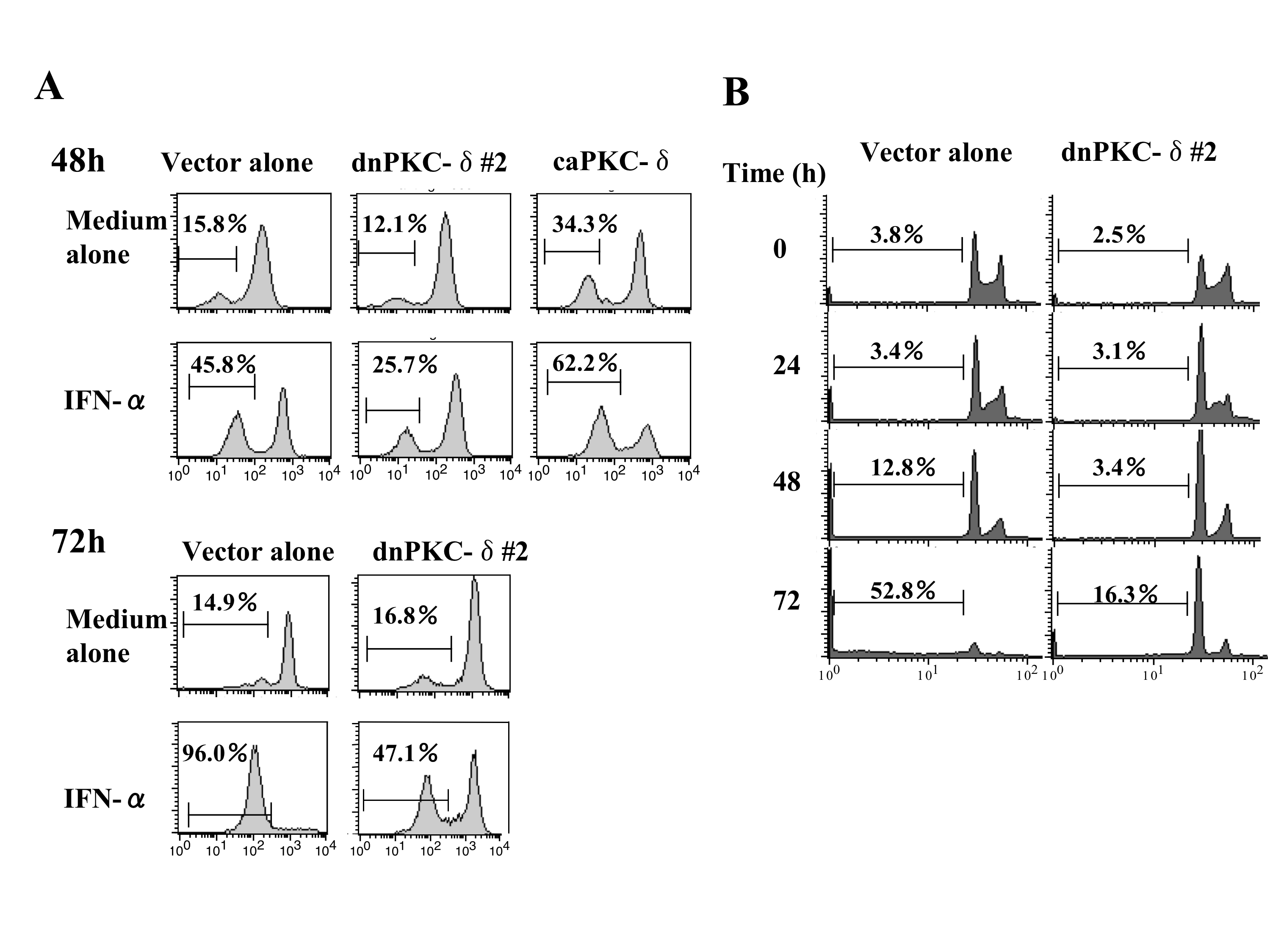

Supplement: Additional file 2 — IFN-α-induced loss of mitochondrial membrane potential and apoptosis are partially abrogated in the dnPKC-δ-expressing cells, while somewhat enhanced in the caPKC-δ-expressing cells. The dnPKC-δ-expressing and caPKC-δ-expressing cells were stimulated with 1,000 U/ml IFN-α for the indicated time periods and assayed as described in Figure legend 4. A, percent cells with low mitochondrial membrane potential; B, percent PI positive cells. [file 1471-2121-13-7-S2.PNG]

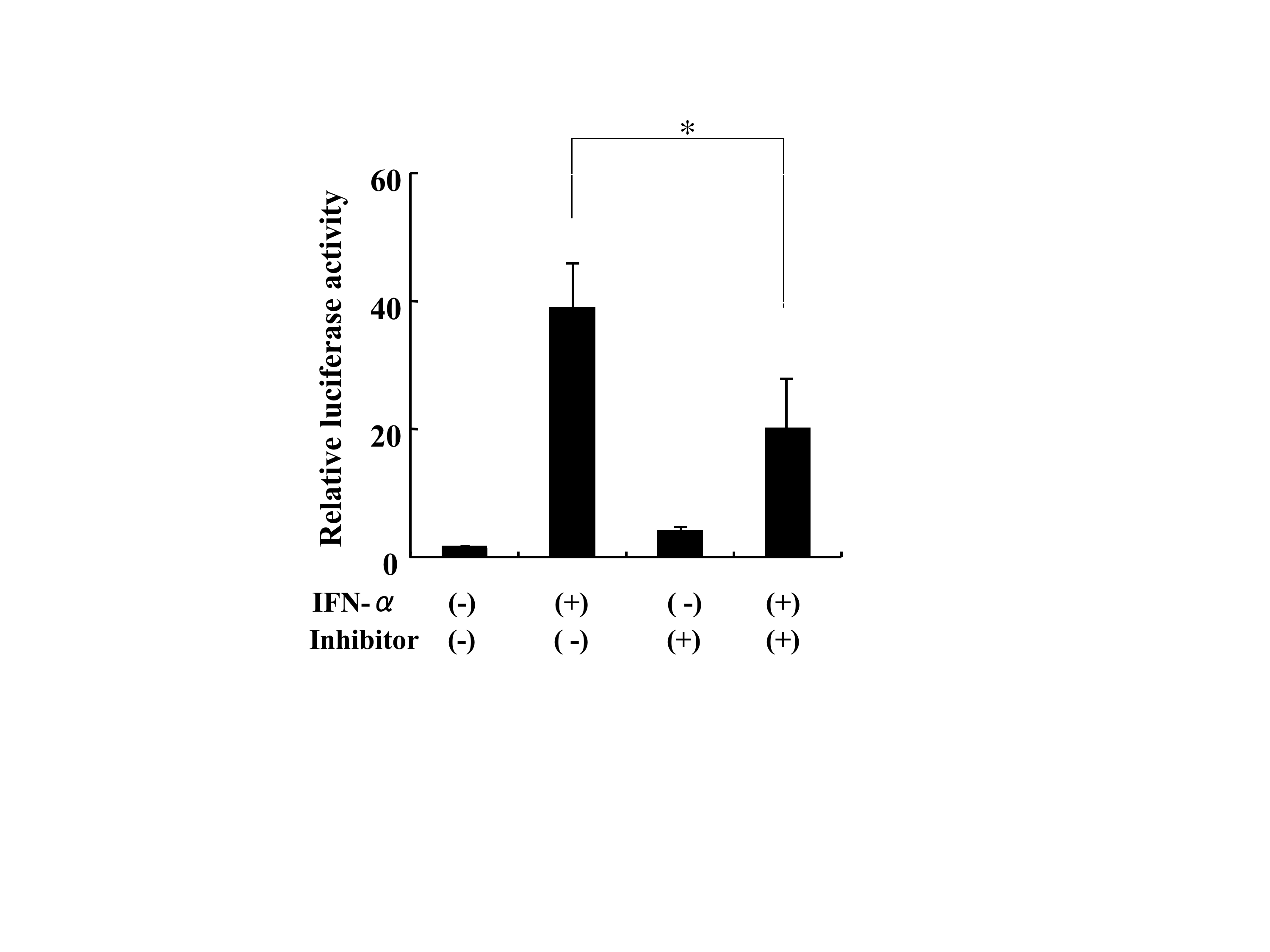

Supplement: Additional file 3 — JNK inhibitor SP-600125 reduces TRAIL promoter activity induced by IFN-α in caPKC-δ-overexpressing Daudi B lymphoma cells. The caPKC-δ-overexpressing Daudi B lymphoma cells were pretreated with SP-600125 for 1 h, stimulated with 1,000 U/ml IFN-α for 24 h, and then assayed for luciferase activity. *Significantly different (p < 0.05) from IFN-α without JNK inhibitor. [file 1471-2121-13-7-S3.PNG]

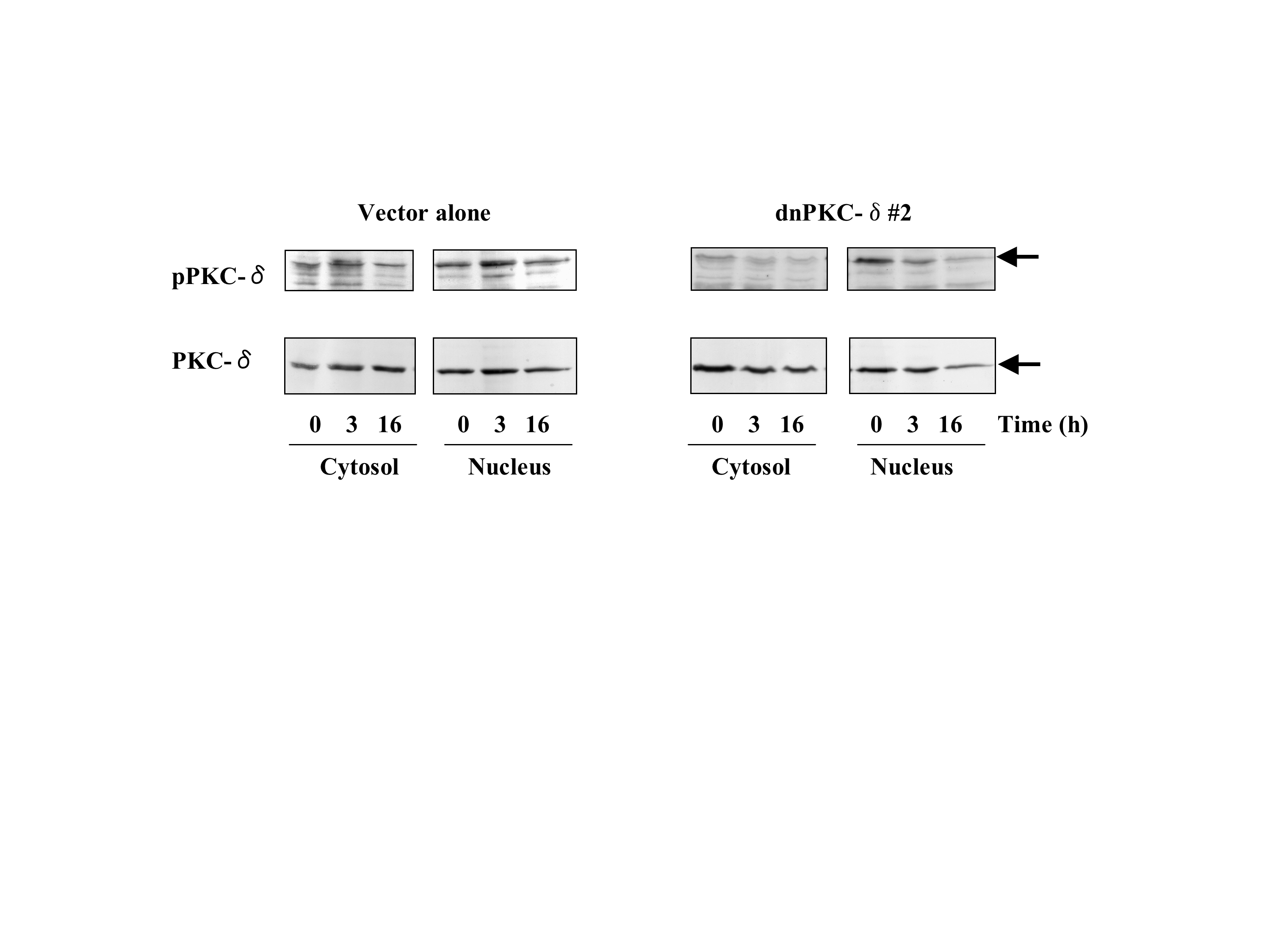

Supplement: Additional file 4 — PKC-δ does not translocate to the nucleus upon IFN-α stimulation. Cells were stimulated with or without 1,000 U/ml IFN-α for the indicated time periods. Cell lysates were fractionated into the cytosolic and nuclear fraction, as described in materials and methods. Samples were assayed for expression levels of total and phospho-PKC-δ by Western blotting. [file 1471-2121-13-7-S4.PNG]
